# Nrf2 Overexpression for the Protective Effect of Skin-Derived Precursors against UV-Induced Damage: Evidence from a Three-Dimensional Skin Model

**DOI:** 10.1155/2019/7021428

**Published:** 2019-10-14

**Authors:** Dehai Xian, Xia Xiong, Jixiang Xu, Li Xian, Qirong Lei, Jing Song, Jianqiao Zhong

**Affiliations:** ^1^Department of Neurobiology, Southwest Medical University, Luzhou 646000, China; ^2^Department of Dermatology, The Affiliated Hospital of Southwest Medical University, Luzhou 646000, China; ^3^Department of Emergency, The Affiliated Hospital of Southwest Medical University, Luzhou 646000, China

## Abstract

**Background:**

Skin photodamage is associated with ultraviolet- (UV-) induced reactive oxygen species (ROS) overproduction and nuclear factor erythroid 2-related factor 2 (Nrf2) inactivation. In our previous study, skin-derived precursors (SKPs) were shown to ameliorate a UV-induced damage in mice, probably through Nrf2 activation and ROS scavenging.

**Objective:**

To clarify the mechanism underlying the photoprotective effect of SKPs against UV-induced damage in a three-dimensional (3D) skin model.

**Methods:**

The *Nrf2* gene in SKPs was modified using lentiviral infection, and 3D skin models were reconstructed with keratinocytes and fibroblasts on the basis of type I collagen. Subsequently, these models were divided into the following six groups: normal, model, overexpressed, control, silenced, and negative control groups. Prior to irradiation, respective SKPs were injected into the last four groups. Next, all groups except the normal group were exposed to UVA+UVB. Lastly, the pathological and molecular-biological techniques were employed to determine the parameters. Additionally, LY294002, a PI3K inhibitor, was used to investigate the roles of PI3K/Akt and Nrf2/hemeoxygenase-1 (HO-1) in SKP photoprotection.

**Results:**

Normal 3D skin models appeared as milky-white analogs with a clear, well-arranged histological structure. After the skin was exposed to irradiation, it exhibited cell swelling and a disorganized structure and developed nuclear condensation with numerous apoptotic cells. The expressions of cellular protective genes and Nrf2/HO-1/PI3K/Akt proteins remarkably decreased, which were accompanied by increased oxidative stress and decreased antioxidants (*P* < 0.05). However, these phenomena were reversed by *nrf2*-overexpressing SKPs. The 3D skin in the overexpressed group showed mild swelling, neatly arranged cells, and few apoptotic cells. Cellular protective genes and Nrf2/HO-1/PI3K/Akt proteins were highly expressed, and the oxidative biomarkers were remarkably ameliorated (*P* < 0.05). Nevertheless, the expression of these proteins decreased after LY294002 pretreatment regardless of SKP treatment or not. Meanwhile, there were increases in both UV-induced apoptotic cells and ROS level accompanied with SOD and GPX decrease in the presence of LY294002.

**Conclusions:**

Evidence from the 3D skin model demonstrates that the protection of SKPs against UV-mediated damage is primarily via the PI3K/Akt-mediated activation of the Nrf2/HO-1 pathway, indicating that SKPs may be a promising candidate for the treatment of photodermatoses.

## 1. Introduction

Overexposure to ultraviolet (UV) light is a causative factor of skin photodamage, characterized by acute effects (such as erythema and sunburn) or chronic effects (such as photoaging and cutaneous tumors) [1, 2]. The photodamaged skin may adversely affect the quality of life in individuals. Thus, the prevention of photodamage is a key research area and much effort has been made for developing effective photoprotectants on the basis of the molecular mechanisms of photodamage. Skin photodamage is initiated by the UV radiation-induced overproduction of reactive oxygen species (ROS) [3–5]. Under normal physiological condition, a slight increase in ROS could induce oxidative stress reaction in the skin [3, 5]; in response, the transcription factor nuclear factor erythroid 2-related factor 2 (Nrf2) is activated under the action of PI3K/Akt, then upregulates hemeoxygenase-1 (HO-1), and further takes part in this process [6]; by orchestrating cellular defense mechanisms, including DNA repair, phase-II detoxification, antioxidant response, and inflammatory signaling, Nrf2 protects the skin cells from UV insult and ameliorates the photooxidative damage [7, 8]. However, excessive ROS generation, especially in photodamage, may inactivate Nrf2, exacerbate the cellular cytotoxicity, and lead to oxidative damage in the skin. Recent studies have demonstrated the protective effect of Nrf2-mediated gene expression on skin photodamage; Nrf2 activation was shown to protect the skin cells against solar UV-induced cytotoxicity and cell damage [9–11]. This indicates that Nrf2 activation is a promising therapeutic target for skin diseases caused by UV radiation.

Although several natural and synthetic pharmacological agents (e.g., procyanidins, rheum, vitamin E, and vitamin C) confer an effect of antiphotooxidative stress and promise a Nrf2-dependent protection against UV-induced damage, most of these agents have some degree of undesirable characteristics, such as transient effects and fast metabolism. Recent years have witnessed a widespread use of the stem cell therapy in several medical disciplines. The pluripotent properties of stem cells, with their ability to deliver high proliferation and self-renewal, make them effective for the treatment of tissue damage and injury. Skin-derived precursor cells (SKPs), adult stem cells of dermal origin, are shown to be superior to other stem cells in the treatment of cutaneous disorders, skin regeneration, and wound healing [12–14]. In our previous study, SKPs were found to strongly resist UVB-induced apoptosis and DNA damage via the upregulation of Bcl-2 and Nrf2 [15]. On the basis of these findings, we conducted an *in vivo* study on hairless mice for assessing the protective role of SKPs in skin photodamage. The results showed that SKPs transplanted to the mouse skin alleviated the damage induced by solar-simulated radiation, possibly via the activation of Nrf2 and its signaling pathway [16]. Besides, we recently established a normal three-dimensional (3D) skin equivalent and photodamaged 3D skin model *in vitro* and eventually confirmed the role of Nrf2 in the 3D skin photodamage [17]; meanwhile, SKPs were firstly injected into the photodamaged 3D skin model *in vitro*, having results revealing the effect of SKPs against the UV-induced 3D skin damage [18]. For clarifying the exact mechanism underlying the protective effect of SKPs against skin photodamage, we performed an *in vitro* study using the photodamaged 3D skin models and *nrf2* gene-modified SKPs as well as determining the components of Nrf2/HO-1 and PI3K/Akt pathways.

## 2. Materials and Methods

### 2.1. Human Sample and Ethics Statement

Human samples were obtained from the foreskin of pediatric patients (aged 3–5 years) who underwent circumcision after obtaining consent from the parents of the donors. The study was conducted after obtaining the informed consent and approval of the Ethics Committee of the Affiliated Hospital of Southwest Medical University (permit number: 20140366A).

### 2.2. Isolation and Culture of Cells

SKPs were isolated from the infant foreskin as previously described [16]. SKPs were cultured in SKP medium (3 : 1 DMEM/F12 containing 20 ng/mL EGF, 40 ng/mL FGF2, 2% B27 supplement, and 0.1% penicillin/streptomycin; Invitrogen, Carlsbad, CA, USA) and incubated at 37°C/5% CO_2_ in a humidified atmosphere.

For reconstructing the 3D skin models, fibroblasts (FBs) and keratinocytes (KC) were isolated from the dermis or epidermis of the foreskin and cultured as described elsewhere [17]. FBs were cultured in the DMEM medium plus 10% fetal bovine serum containing 0.1% penicillin/streptomycin and 0.25 *μ*g/mL amphotericin B (Invitrogen, Carlsbad, CA, USA). KC were cultured in low-calcium, serum-free keratinocyte-SFM containing 0.1% penicillin/streptomycin and 0.25 *μ*g/mL amphotericin B (Invitrogen, Carlsbad, CA, USA).

All these cells have been identified in our previous studies [16, 17].

### 2.3. Construction of Lentivirus Vectors

Recombinant lentiviruses used for overexpressing and silencing *nrf2* were purchased from GeneChem (Shanghai, China). LentiORF lentiviral particles (GV358, Ubi-MCS-3FLAG-SV40-ERFP-IRES-puromycin, LVKL25280-2) and lenti-shRNA vectors (GV248, hU6-MCS-ubiquitin-EGFP-IRES-puromycin, LVpFU-GW-007) were constructed, packed, and purified by GeneChem (Shanghai, China). SKPs were transfected with lentivirus-containing lenti-RFP control (lenti-C or lenti-C-SKPs), lenti-overexpressed *nrf2* (lenti-*nrf2* or *nrf2-over-*SKPs), lenti-silenced *nrf2* (lenti-shRNA-*nrf2*, lenti-sh*nrf2*, or *si-nrf2-*SKPs), and lenti-shRNA-GFP-negative control (lenti-shNC or lenti-shNC-SKPs). Next, SKPs were incubated at 37°C/5% CO_2_ and selected using puromycin. Lastly, puromycin-resistant cells were allowed to grow in the SKP medium for further experiments.

### 2.4. Reconstruction of the 3D Skin Model

Human 3D skin models were generated as previously described [17, 19]. All manipulations were performed on ice for preventing premature collagen polymerization. Solution of collagen I extracted from rat tail (HONGYUE CHUANGXIN Technology Co. Ltd., Beijing, China; BD Biosciences, Bedford, MA; 3.9 mg/mL) was mixed with 5 times DMEM media. Next, 1 mol/L NaOH was quickly added to adjust the pH of the solution to 7.2–7.4 and to prepare a collagen mixture solution. Immediately, 1 × 10^5^ cells/mL FBs were mixed with 1 mL of collagen mixture solution to avoid bubble formation. After incubation at 37°C for 2 h, polymerized collagen fibrils (collagen gel) were formed and immersed in serum-free keratinocyte-SFM culture medium for 3–4 days for generating collagen gel dermis. Next, the collagen gels were transferred to the transwell inserts housed in a 6-well cluster plate (BD Biosciences, Bedford, MA; diameter 2.5 cm; pore size 3.0 mm) and seeded with human primary KC at a density of 5 × 10^5^ cells/cm^2^. After 3-day soaked culture in serum-free keratinocyte-SFM, the collagen gels continued to be cultured for 7 days at the air-liquid interface. The medium was changed every two days and the collagen gels were observed daily under an inverted microscope. To confirm the characteristics, the 3D skin models or reconstructed skin equivalents were collected for detection of CK10 and vimentin markers by histological and immunohistochemical (IHC) examinations.

### 2.5. Application of SKPs to Photodamaged 3D Skin Models

#### 2.5.1. Group Division

The reconstructed 3D skin models were randomly divided into the following six groups: normal group (no intervention), model group (UVA+UVB), overexpressed group (*nrf2-over-*SKPs+UVA+UVB), control group (lenti-C-SKPs+UVA+UVB), silenced group (*si-nrf2-*SKPs+UVA+UVB), and negative control group (lenti-shNC-SKPs+UVA+UVB). Each group was examined 24 h after UV irradiation.

#### 2.5.2. SKP Injection into 3D Skin Models

Prior to the UV irradiation, *nrf2-over-*SKPs, lenti-C-SKPs, *si-nrf2-*SKPs, and lenti-shNC-SKPs were injected into the overexpressed group, control group, silenced group, and negative control group, respectively. However, the normal group received no treatment. Before injection, cells were resuspended in HBSS at a concentration of 1 × 10^4^ cells/*μ*L. Approximately 100 *μ*L of cell suspension was injected per cm^2^ 3D skin by using a gauge needle. Then, the skin models with the dermal side immersed in the medium were cultured for 24 h at the air-liquid interface. In a separate experiment, 3D skin equivalents were pretreated with or without LY294002 (10 *μ*M, a specific inhibitor of PI3K, Sigma-Aldrich) for one hour prior to *nrf2-over-*SKPs injection.

#### 2.5.3. UV Irradiation

Twenty-four hours following injection of SKPs, the 3D skin models were washed twice with HBSS and irradiated once with UVA at 3 J/cm^2^ and UVB at 90 mJ/cm^2^ for 60 seconds using a solar simulator (SUV1000, 290–400 nm, Shanghai Sigma High-tech Co. Ltd., Shanghai, China). The spectrum of lamp emission comprised 95% UVA and 5% UVB. The distance from the lamps to the models was 20 cm, and the plate was kept open during irradiation. Subsequently, these models continued to be cultured in the SKP medium for 24 h. Lastly, the supernatants and skin models were collected for further experiments.

### 2.6. Histology, TUNEL Staining, and IHC Analysis

Reconstructed skin equivalents from the above groups were in turn fixed in 4% paraformaldehyde, embedded in paraffin, and cut into 6 *μ*m serial slices. Histological features were determined using hematoxylin and eosin (H&E) staining. Apoptotic cells were detected using TUNEL staining kit (Abcam, UK) in accordance with the manufacturer's instructions. Cells with the positive expressions of vimentin and CK10 (Abcam, UK) were measured using IHC analysis. Positively stained cells were counted in five randomly selected fields through a double-blind microscopic evaluation by two independent observers. Simultaneously, the survival of different SKPs was tracked using fluorescent labeling.

### 2.7. ELISA for Assessment of the Biomarker of Oxidative Stress

Oxidative stress biomarkers including SOD (SOD1), CAT, GPX, GSH, ROS, and MDA were measured in the supernatants of various groups. ELISA assay kits for SOD, CAT, GPX, and GSH were obtained from Nanjing Jiancheng Bioengineering Institute (Nanjing, China), while those kits for ROS and MDA were supplied by Nanjing SenBeiJia Bio-Technology Co. Ltd. (Nanjing, China). All the parameters were determined using commercial kits in accordance with the manufacturers' instructions.

### 2.8. Real-Time Quantitative Reverse Transcription PCR (qRT-PCR) Analysis

For measuring the mRNA levels of Nrf2 and HO-1, total RNA was extracted from SKPs, whereas total RNA was extracted from the 3D skin models for detecting the mRNA expressions of cellular protective genes, including *nrf2*, *ho-1*, *gpx*, *sod*, *cat*, and *gsh* using the TRIzol RNA extraction kit in accordance with the manufacturer's instructions (Invitrogen, Carlsbad, CA, USA). Target gene expressions were determined relative to those of *gapdh* (internal control). The primers used are listed in Table 1.

### 2.9. Western Blot Analysis

Western blotting was performed as previously described. For the detection of Nrf2 and HO-1 protein levels, total proteins were extracted from the SKPs and 3D skin models using the lysis and protein loading buffers. The protein concentrations were determined using Pierce BCA Protein Assay. The Bis-Tris Gel system was established, and the primary antibodies against protein nuclear Nrf2 (anti-Nrf2 polyclonal antibody, 1 : 1000, Abcam, UK), HO-1 (anti-HO-1 monoclonal antibody, 1 : 2000, Abcam, UK), phosphorylated PI3K (p-PI3K, anti-PI3K polyclonal antibody, and phospho Y607, 1 : 1000, Abcam, UK), and phosphorylated Akt (p-Akt, anti-Akt polyclonal antibody, and phospho T308, 1 : 500, Abcam, UK) were introduced. After incubation with the secondary antibody (goat anti-rabbit IgG, 1 : 5000; Abcam, UK), signals were detected and analyzed using enhanced chemiluminescence detection system (Bio-Rad Laboratories Inc., CA, USA) and Image software. Anti-*β*-actin polyclonal antibody (1 : 5000; Abcam, UK) or anti-GAPDH polyclonal antibody (1 : 5000; Abcam, UK) was used as internal controls.

### 2.10. Statistical Analysis

Data are presented as mean ± SD and the statistical analysis was performed using SPSS 19.0. Between-group differences were assessed using analysis of variance (ANOVA) or Student's *t*-test. Differences were considered to be statistically significant at *P* < 0.05.

## 3. Results

### 3.1. Characteristics of SKPs Infected with *nrf2*-Expressed Lentivirus

**Figure 1:**
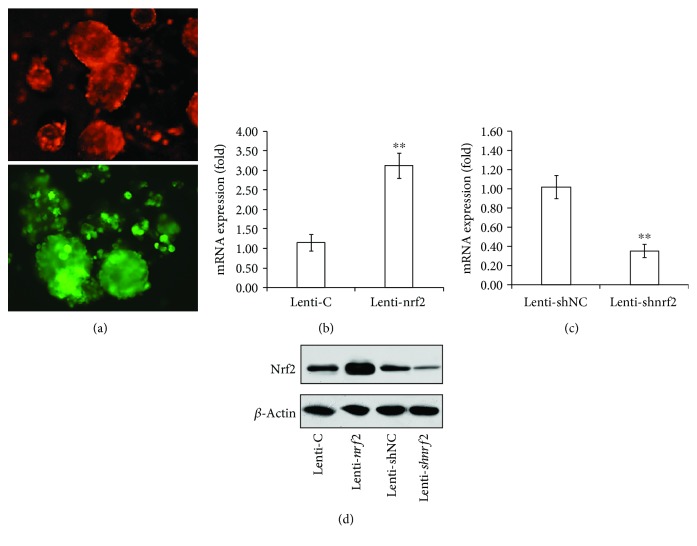
Lentivirus-mediated Nrf2 overexpression and silencing in SKPs: (a) manifestation of SKPs after the *nrf2*-expressed lentivirus infection; *nrf2-over-*SKPs exhibited red fluorescence, whereas *si-nrf2-*SKPs exhibited green fluorescence; (b) mRNA expression of *nrf2* in SKPs with overexpressed expression; (c) mRNA expression of *nrf2* in SKPs with silenced expression; (d) protein expression of Nrf2 in *nrf2-over-*SKPs and *si-nrf2-*SKPs. lenti-C: lenti-RFP control; lenti-*nrf2*: lenti-overexpressed *nrf2*; lenti-shNC: lenti-shRNA-negative control; lenti-sh*nrf2*: lenti-silenced *nrf2*. ^∗∗^*P* < 0.01 (ANOVA).

Twenty-four hours following infection, SKPs exhibited red fluorescence (SKPs with overexpressed *nrf2* expression, *nrf2-over-*SKPs) or green fluorescence (SKPs with silenced *nrf2* expression, *si-nrf2-*SKPs) and grew well with normal morphology (Figure 1(a)). After transfection with lenti-*nrf*2, *nrf2-over-*SKPs showed a significant increase in the mRNA and protein expression of Nrf2 as compared with those in control lenti-C (Figures 1(b) and 1(d), *P* < 0.01). Conversely, shRNA-*nrf*2 remarkably decreased the mRNA and protein expressions of Nrf2 in *si-nrf2-*SKPs as compared with those in lenti-shRNA negative control (Figures 1(c) and 1(d), *P* < 0.01). The results demonstrate a feasibility of overexpression and silencing of Nrf2 that assists the potential application of SKPs in further studies.

### 3.2. Manifestation of Normal Human Skin 3D Model

**Figure 2:**
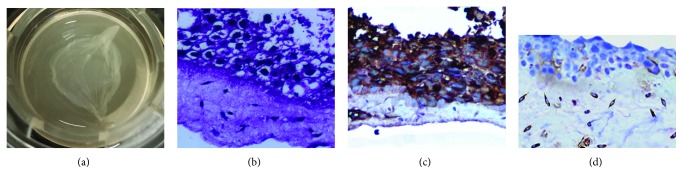
Characteristics of the human skin 3D model or 3D skin equivalent: (a) gross appearance of 3D skin equivalent; (b) the histology of 3D skin equivalent covering the epidermis and dermis (×400); (c) CK10-positive keratinocytes display brown-yellow staining in the epidermis (×400); (d) vimentin-positive FBs show brownish staining in the dermis (×400).

After a two-week culture, a 3D full-thickness living skin model (3D skin equivalent) was successfully constructed using primary epidermal KC and dermal FBs. Its appearance was similar to that of natural skin; it appeared as a milky-white membrane-like structure that exhibited good elasticity and high tensile resistance (Figure 2(a)). Histologically, the 3D skin equivalent contained the epidermis and dermis. The epidermis was clearly stratified and revealed a distinct layered structure; KC arranged tightly with large round and deep-staining nuclei (Figure 2(b)) and positively expressed CK10 (Figure 2(c)). In the dermis, the collagen fibers were stained, revealing a uniformly narrow bright-reddish color, in which fusiform or irregular-shaped FBs were scattered with the oval-shaped nuclei (Figure 2(b)) and positive expression of vimentin (Figure 2(d)).

### 3.3. Nrf2 overexpression in SKPs improved the UV-mediated 3D skin histological structure.

**Figure 3:**
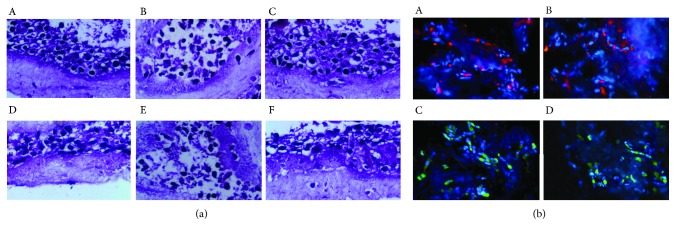
Protective effect of *nrf2-over-*SKPs against UV-mediated 3D skin histological alteration. (a) Sections of the 3D skin model from different groups were stained with H&E (×400). A: normal group; B: model group; C: overexpressed group; D: control group; E: silenced group; F: negative control group. H&E: hematoxylin and eosin. (b) Survival of different SKPs in 3D skin equivalents (×400). A: *nrf2-over-*SKPs injected into the 3D skin emitted red fluorescence in the overexpressed group; B: lenti-C-SKPs injected into the 3D skin emitted red fluorescence in the control group; C: *si-nrf2-*SKPs injected into the 3D skin emitted green fluorescence in the silenced group; D: lenti-shNC-SKPs injected into the 3D skin emitted green fluorescence in the negative control group.

Exposure of the 3D skin equivalent to UVA+UVB radiation resulted in typical pathological alterations, that is, epidermal edema, disorganization of arrangement, gap widening, epidermal cell degeneration with shrunken hyperchromatic nuclei, and uniform reddish cytoplasm (Figure 3(a), B). Interestingly, these changes were reversed with the pretreatment of *nrf2-over-*SKPs, and the histological characteristics were almost similar to those in the normal group (Figure 3(a), C and A). Conversely, *si-nrf2-*SKPs failed to make this condition better as severe pathological derangement of the 3D skin structure was observed in the silenced group, similar to that in the model group (Figure 3(a), E). Although both the control and negative control groups showed an obvious improvement compared with that in the model group, these alterations were inferior to those in the overexpressed group (Figure 3(a), D and F).

All the four SKPs survived well after injection into 3D skin models. On fluorescent microscopy, *nrf2-over-*SKPs and lenti-C-SKPs exhibited red fluorescence in the overexpressed and control groups, respectively, whereas *si-nrf2-*SKPs showed green fluorescence protein in the silenced group. Besides, lenti-shNC-SKPs displayed green fluorescence protein in the negative control group (Figure 3(b)).

### 3.4. Nrf2 Overexpression in SKPs Prevented/Reduced UV-Mediated Apoptosis

**Figure 4:**
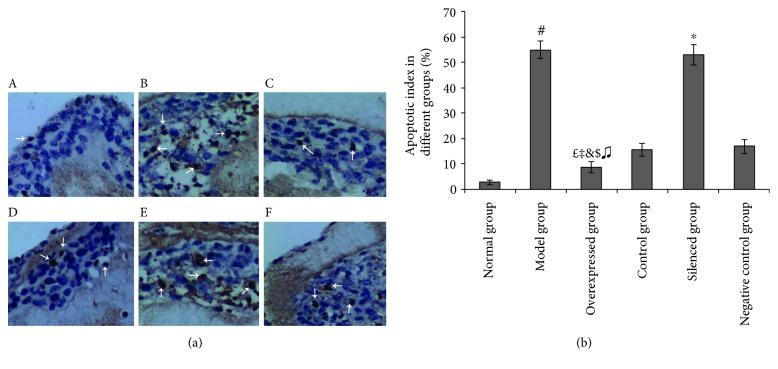
Inhibitory effect of *nrf2-over-*SKPs on UV-induced apoptosis in 3D skin equivalent. (a) Apoptotic cells of 3D skin equivalents were analyzed by TUNEL staining (×400). A: normal group; B: model group; C: overexpressed group; D: control group; E: silenced group; F: negative control group. In the TUNEL assay, the nuclei of apoptotic cells were stained brown. White arrows indicate TUNEL-positive cells. (b) Quantitative analysis of TUNEL-positive cells in different groups. ^#^*P* < 0.01 versus the normal group; ^*£*^*P* < 0.01 versus the normal group; ^‡^*P* < 0.01 versus the model group; ^&^*P* < 0.01 versus the control group; ^$^*P* < 0.01 versus the silenced group; ^♫^*P* < 0.01 versus negative the control group; ^∗^*P* < 0.01 versus the negative control group (ANOVA).

Scarce apoptotic changes were observed in the normal group. TUNEL staining analysis revealed that UV exposure significantly promoted apoptosis of cutaneous cells, which was remarkably attenuated by *nrf2-over-*SKPs (*P* < 0.01). Although the apoptotic cells in the control and negative control groups decreased in number, these were obviously inferior to those in the overexpressed group (*P* < 0.01). Conversely, *si-nrf2-*SKPs failed to ameliorate UV-induced apoptosis. TUNEL-positive cells in the overexpressed group were significantly fewer than those in the other groups, except in the normal group (*P* < 0.01). TUNEL-positive cells in the silenced group significantly outnumbered those in the negative control group (*P* < 0.01). However, no significant difference was found between the model and silenced groups (*P* > 0.05) (Figure 4).

### 3.5. Nrf2 Overexpression in SKPs Attenuated UV-Induced Oxidative Stress Damage

UV irradiation markedly lowered the activities of superoxide dismutase (SOD), catalase (CAT), and glutathione peroxidase (GPX) and the levels of reduced glutathione (GSH), whereas UV irradiation remarkably elevated the levels of malondialdehyde (MDA) and ROS (*P* < 0.01; Table 2). Nevertheless, *nrf2-over-*SKP treatment reversed these conditions, leading to a significant increase in SOD, CAT, GPX, and GSH and a significant decrease in ROS and MDA (*P* < 0.01), which were superior in comparison with the control group (*P* < 0.01). However, few improvements were observed using the *si-nrf2-*SKPs treatment but not by lenti-shNC-SKPs. Indeed, some deterioration was noticeable in the silenced group as compared with that in the negative control group (*P* < 0.05).

### 3.6. Nrf2 Overexpression in SKPs Promoted the Expressions of Cellular Protective Genes

**Figure 5:**
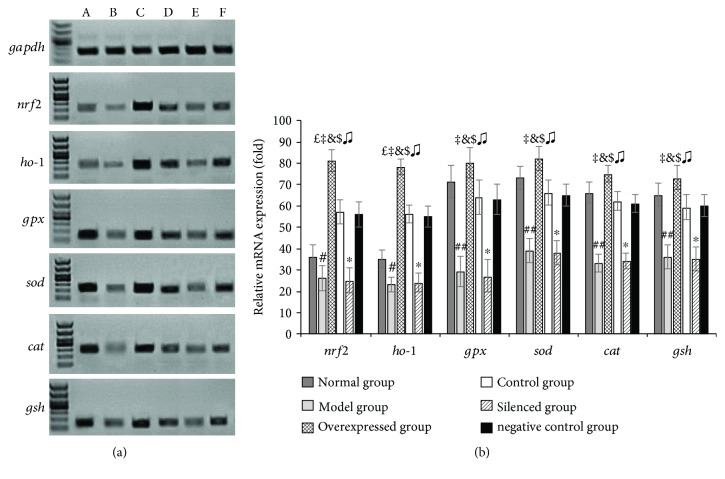
Effect of *nrf2-over-*SKPs on expression of cellular protective genes in 3D skin equivalent. (a) Manifestation of cellular protective genes on agarose gel electrophotogram. A: normal group; B: model group; C: overexpressed group; D: control group; E: silenced group; F: negative control group. (b) Relative mRNA levels of cellular protective genes in different groups. ^#^*P* < 0.05 and ^##^*P* < 0.01 versus the normal group; ^*£*^*P* < 0.01 versus the normal group; ^‡^*P* < 0.01 versus the model group; ^&^*P* < 0.01 versus the control group; ^$^*P* < 0.01 versus the silenced group; ^♫^*P* < 0.01 versus the negative control group; ^∗^*P* < 0.01 versus the negative control group (ANOVA).

On UV irradiation of the 3D skin equivalents, varying degrees of drop in the mRNA levels of *nrf2*, *ho-1*, *gpx*, *sod*, *cat*, and *gsh* were observed (*P* < 0.05) (Figure 5). However, these gene expressions were significantly upregulated in the control and overexpressed groups after pretreatment with different SKPs, the overexpressed group by *nrf2-over-*SKP treatment in particular (*P* < 0.01). Conversely, the *si-nrf2-*SKP treatment had little effect on gene expressions in the UV-damaged 3D skin. The levels of cellular protective genes in the silenced group were significantly lower than those in the negative control and overexpressed groups (*P* < 0.01) (Figure 5).

### 3.7. Nrf2 Overexpression in SKPs Stimulated the Nrf2/HO-1 and PI3K/Akt Pathways

**Figure 6:**
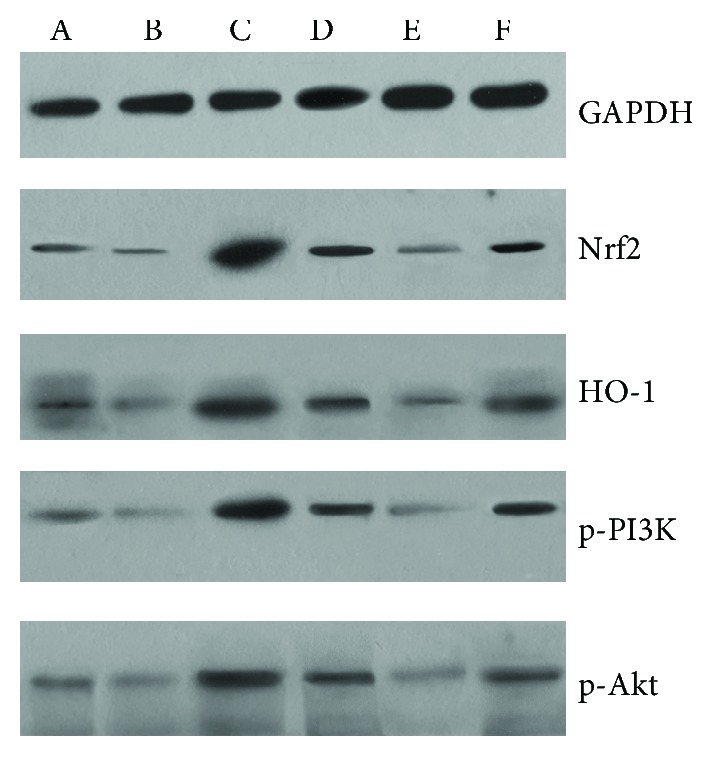
Effect of *nrf2-over-*SKPs on the expression of components of Nrf2/HO-1 and PI3K/Akt pathways in 3D skin equivalent: a: normal group; b: model group; c: overexpressed group; d: control group; e: silenced group; f: negative control group.

To unravel the mechanism of SKPs protecting the skin against UV-induced damage, the components of the Nrf2/HO-1 and PI3K/Akt pathways were investigated. The proteins, including Nrf2, HO-1, PI3K, and Akt, were weakly expressed in the normal 3D skin equivalent. After exposure to a high dose of UV radiation, Nrf2 and HO-1 expressions in the 3D skin decreased; however, *nrf2-over-*SKP treatment notably increased Nrf2 and HO-1 protein expressions in the UV-damaged 3D skin (Figure 6). Similar changes were observed in PI3K and Akt phosphorylation proteins, that is, UV irradiation inhibited the phosphorylation of PI3K and Akt proteins, whereas *nrf2-over-*SKPs stimulated and promoted the expressions of PI3K and Akt phosphorylation proteins. The protein bands in the overexpressed group were remarkably stronger than those in the control group. On the contrary, *si-nrf2-*SKPs were ineffective in increasing these proteins in the UV-damaged 3D skin. The silenced group showed significantly low expression of Nrf2, HO-1, PI3K, and Akt, as compared with those in the negative control and overexpressed groups (Figure 6).

**Figure 7:**
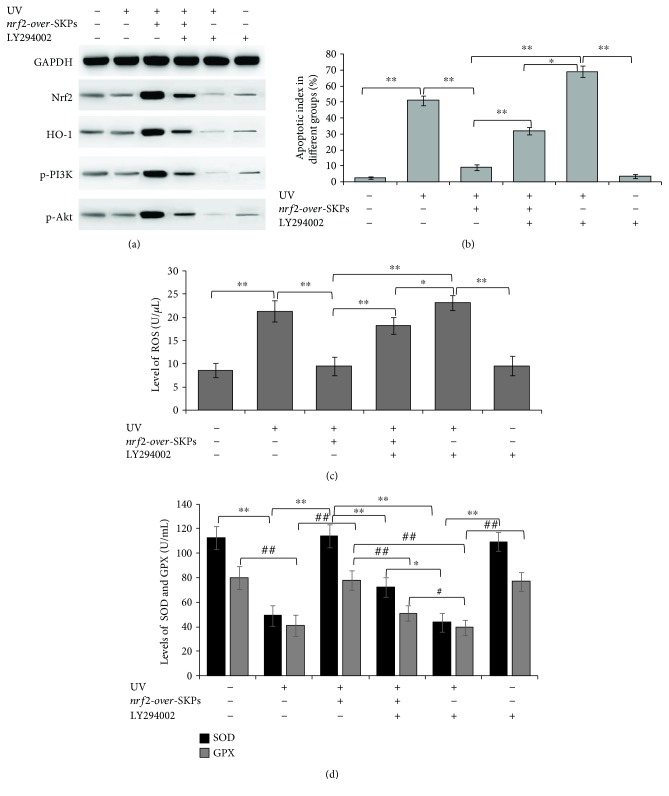
PI3K/Akt inhibitor LY294002 attenuated the photoprotection of *nrf2-over-*SKPs in 3D skin equivalent. (a) The protein levels of Nrf2, HO-1, p-PI3K, and p-Akt in 3D skin equivalents by Western blot analysis; (b) quantitative analysis of apoptotic cells in 3D skin equivalents by TUNEL staining. ^∗^*P* < 0.05 and ^∗∗^*P* < 0.01; (c) ELISA assay for ROS expression in the supernatants of 3D skin equivalents. ^∗^*P* < 0.05 and ^∗∗^*P* < 0.01; (d) ELISA assay for SOD and GPX expressions in the supernatants of 3D skin equivalents. ^∗^*P* < 0.05, ^∗∗^*P* < 0.01, ^#^*P* < 0.05, and ^##^*P* < 0.01.

Further to confirm the specific role of PI3K/Akt in the activation of the Nrf2/HO-1 pathway, the PI3K inhibitor (LY294002) was employed. Although *nrf2-over-*SKPs greatly enhanced the expression of components of the Nrf2/HO-1 and PI3K/Akt pathways, their effect was attenuated in the presence of LY294002. After pretreatment with LY294002 and *nrf2-over-*SKPs as well as exposure to UV, the expressions of Nrf2, HO-1, p-PI3K, and p-Akt in the 3D skin apparently weakened compared with those pretreated with *nrf2-over-*SKPs and UV (Figure 7(a)), that is, the phosphorylation of Nrf2, HO-1, PI3K, and Akt was inhibited by LY294002 regardless of *nrf2-over-*SKP intervention or not. At the same time, the UV-induced apoptotic cells in 3D skin equivalents with LY294002 greatly outnumbered those without the PI3K inhibitor (*P* < 0.01), even in the presence of *nrf2-over-*SKPs (Figure 7(b)). A similar change occurred in ROS, namely, ROS expression in supernatants ascended after LY294002 treatment (*P* < 0.05) (Figure 7(c)); conversely, the levels of SOD and GPX descended (*P* < 0.05), those only with UV and LY294002 intervention in particular (*P* < 0.01) (Figure 7(d)).

## 4. Discussion

In the present study, we successfully uncovered the mechanism underlying the protective effect of SKPs against the UV-induced damage in a 3D skin model. First, SKPs were gene modified and injected into a well-constructed 3D skin model, followed by the 3D skin exposure to UV. Histologically, *nrf2* gene-overexpressing SKPs were extremely effective against the UV-damaged 3D skin, whereas the *nrf2* gene-silenced SKPs were ineffective. *Nrf2* gene-overexpressing SKPs remarkably reduced the UV-induced apoptosis of cutaneous cells in the 3D skin model, which decreased ROS and MDA and increased GPX, SOD, and CAT. Lastly, the 3D skin model, treated with *nrf2* gene-overexpressing SKPs in the absence of LY294002, showed high expression levels of cellular protective genes and the Nrf2, HO-1, p-PI3K, and p-Akt proteins; but the phosphorylated levels of those proteins remarkably dropped in the presence of the PI3K inhibitor (LY294002), accompanied with the enhancement of ROS and the fall of SOD and GPX. These findings indicate that SKPs target Nrf2 for protection against the UV-induced damage via the activation of the Nrf2/HO-1 and PI3K/Akt pathways.

To simulate skin photodamage, we established a skin photodamage model *in vitro*. Although various models of the UV-induced damage on the basis of cells, tissue culture, and animals have been employed, these techniques have some limitations, such as high costs of application and inaccurate simulation of native skin biology and structure [20–23]. The 3D skin equivalent has addressed these limitations and appears to be an ideal model that replicates the human skin [24, 25]. The 3D skin equivalents were introduced to establish a photodamage model *in vitro* in a previous study [17]. On the basis of our previous experience [17], milky-white analogs of the human skin were successfully harvested with positive expressions of the special epidermal and dermal markers containing CK10 and vimentin. Following mixed irradiation of these skin equivalents with a single-dose of UVA (3 J/cm^2^)+UVB (90 mJ/cm^2^), we observed the typical pathological changes associated with photodamage; these findings were consistent with those in previous reports that documented similar changes (epidermal edema, shrunken cells, and nuclear concentration (sunburn cells)) in an *in vitro* model of the UV-induced skin damage [17, 26]. This indicated a successful establishment of the 3D photodamage models in our study, which laid a firm foundation for further studies.

Our previous studies demonstrated an excellent *in vitro* and *in vivo* protective efficacy of SKPs against UV-induced apoptosis or damage, likely mediated through the activation of Nrf2 [16]. The photopreventive effect of Nrf2 gene activation has been confirmed by several studies [6, 8, 27–29]. Additionally, the exogenous or endogenous constitutive activation of Nrf2 has been developed as a new strategy for skin photoprotection. For clarifying the underlying mechanisms, we overexpressed and silenced the *nrf2* gene of SKPs and injected the gene-modified SKPs into the well-established 3D-photodamaged skin model. Predictably, SKPs with *nrf2* overexpression significantly ameliorated the UV-induced structural derangement, decreased the UV-induced apoptosis, and reversed the state of oxidative stress, excelling in the control group by lenti-C-SKP treatment. Interestingly, this effect was not observed in the absence of the *nrf2* gene. SKPs with silenced *nrf2* ineffectively worked on 3D photodamaged skin. Conversely, SKPs with non-targeting-silenced *nrf2* showed a protective effect against the UV-induced 3D skin damage. The pathological alterations of 3D skin models in the negative control and control groups improved in comparison with those in the silenced group; however, the effect was less remarkable than that in the overexpressed group. These results are consistent with those of previous studies as follows. In a study by Li and colleagues, Nrf2 overexpression in adipose-derived stem cells greatly accelerated skin wound healing by relieving the oxidative damage and promoting the proliferation of endothelial progenitor cells [30]. Yang et al. reported that bone marrow mesenchymal stem cells significantly alleviated the oxidative damage and decreased apoptosis in a rat model with spinal cord injury, through activation of the Nrf2 pathway [31]. Likewise, in a study by Chen and colleagues, adipose-derived stem cells were shown to modulate the oxidative stress in *nrf2* gene-modified mice via the activation of the Nrf2-mediated pathway [32]. Our present findings confirm that SKPs were effective in the UV-induced 3D skin model in the presence of Nrf2 but not in the absence of Nrf2. These findings indicate that Nrf2 is crucial in mediating the effect of SKPs against skin photodamage. However, the specific mechanism by which Nrf2 overexpression in SKPs is employed in protecting against photodamage was unclear.

Therefore, the Nrf2 pathway and its upstream/downstream factors were investigated. The Nrf2 pathway is deemed to be one of the main ROS signaling pathways. Nrf2 translocation upregulates the transcription of its downstream factor (e.g., HO-1 gene) to fight oxidative stress. Under normal physiological conditions, Nrf2 is inactively located in the cytoplasm by binding to its repressor Kelch-like ECH-associated protein 1 (Keap1) that impedes Nrf2 into the nucleus and promotes it degradation [33]. This, maybe, is the reason that nucleus Nrf2/HO-1 was at a low level in the normal group in our study. Upon oxidative stimulation like a moderate UV irradiation, Nrf2 dissociates from Keapl into the nucleus and then binds to the antioxidant response element (ARE) in the promoter region to induce the transcription of cytoprotective genes and antioxidants [34]. However, excessive ROS generated from high-dose UV fail to perform Nrf2 activation and make it insufficient, thereby leading to its upstream/downstream factor inertia and the irreversible cell injury [35], which may explain why the expression of Nrf2/HO-1 and PI3K/Akt proteins significantly decreased in the model group in the current study. Thus, the activation of Nrf2 signaling as well as its upstream/downstream factors, e.g., PI3K, Akt, and HO-1, is quite crucial to the antioxidant and photoprotective activities, which could facilitate cell survival, promote redox balance, and resist oxidative stress [36]. Interestingly, in the present experiment, the *nrf2-over-*SKP-treated 3D skin showed high expressions of Nrf2, HO-1, PI3K, and Akt proteins, which were accompanied with an increase in SOD, CAT, GPX, and GSH levels and decrease in ROS and MDA levels. Moreover, the mRNA expression of cellular protective genes, including *nrf2*, *ho-1*, *gpx*, *sod*, *cat*, and *gsh*, remarkably rose in the overexpressed group. These findings confirm that Nrf2-overexpressing SKPs not only produce high-level antioxidants (e.g., HO-1, SOD, CAT, GPX, and GSH) and eliminate oxides but also upregulate the expressions of cellular protective genes through the activation of the Nrf2 signaling pathway. Similar results were reported by Mohammadzadeh et al. and Calkins et al.; they found that Nrf2 overexpression in stem cells enhanced the activities of numerous antioxidants, such as HO-1 and SOD, which protected cells/tissue from the oxidative insult [37, 38].

As an important regulator of redox homeostasis, Nrf2 protects cells/tissues from oxidative stress by regulating several crucial signaling pathways, such as HO-1, FTH1, and NQO1. Among those pathways, Nrf2/HO-1 signaling plays a prominent role. HO-1 (a key downstream protein of Nrf2) belongs to a larger family of stress proteins; the activated HO-1 responds against the oxidative stress state via the PI3K/Akt-mediated Nrf2/HO-1 signaling pathway. As the upstream of the Nrf2/HO-1, PI3K/AKT pathway widely exists in cells to promote cell survival and regulate metabolic homeostasis. The activation of the PI3K/Akt pathway benefits to Nrf2 translocation and HO-1 synthesis. Several studies have demonstrated that PI3K/Akt plays a critical role in the modulation of HO-1 activation and Nrf2/HO-1 antioxidation. The stimulation of PI3K/Akt by an agonist was shown to promote HO-1 expression in a Nrf2-dependent manner and protect cells from an oxidative insult [39–41]. On the contrary, the suppression of PI3K/Akt by an inhibitor prevented the HO-1 activation and Nrf2/HO-1 response to oxidative stress [42]. Our results coincided with the above findings. Interestingly, we discovered that the levels of Nrf2, HO-1, PI3K, and Akt proteins apparently increased in *nrf2-over-*SKP presence and LY294002 absence, whereas those proteins levels were partly downregulated by LY294002 even in the presence of *nrf2-over-*SKPs. These indicated that the PI3K/Akt pathway was involved in the activation of the Nrf2/HO-1 pathway. The outcomes from the present study demonstrated that Nrf2-overexpressing SKPs promoted the Nrf2 translocation, stimulated Nrf2/HO-1 activation, and enhanced the expressions of PI3K and Akt proteins for protection against the UV-induced damage, suggesting that Nrf2/HO-1 activation is involved in the protective effect of SKPs against the UV-induced skin damage via the stimulation of the PI3K/Akt signaling pathway.

## 5. Conclusions

In conclusion, our findings reveal that SKPs, *nrf2*-overexpressing SKPs in particular, could ameliorate UV-induced damage in a 3D skin model and Nrf2 is a key target mediating the protective effect of SKPs against photooxidative damage. The underlying mechanism of the protective effect of SKPs may involve the activation of the Nrf2/HO-1 and PI3K/Akt signaling pathways. Moreover, SKPs activate the Nrf2/HO-1 pathway mostly via stimulating the PI3K/Akt pathway. Therefore, the modulation of the Nrf2 system would help in enhancing the effect of SKPs against the UV-mediated damage, which implies a potential remedial value of SKPs in the management of skin photodamage.

## Figures and Tables

**Table 1:** The primers of cellular protective genes.

Genes	Forward	Reverse
*gapdh*	5′-TGGTGAAGGTCGGTGTGAAC-3′	5′-GCTCCTGGAAGATGGTGATGG-3′
*nrf2*	5′-CTCCTATGCGTGAATCCCAATG-3′	5′-GGCGGCGACTTTATTCTTACC-3′
*ho-1*	5′-CTCCTATGCGTGAATCCCAATG-3′	5′-GGCAAGATTCTCCCTTACAGA-3′
*gpx*	5′-CCCCAAGTACATCATTTGGTC-3′	5′-GGGACAGCAGGGTTTCTATG-3′
*sod*	5′-TGTACCAGTGCAGGACCTCAT-3′	5′-GCCCAAGTCATCTTGTTTCTC-3′
*cat*	5′-TGTGAACTGTCCCTACCGC-3′	5′-TAGAATGTCCGCACCTGAG-3′
*gsh*	5′-GGATGAGGACAGCGTTTAC-3′	5′-ACCAGATTTTCACCTGCTT-3′

**Table 2:** Effect of SKPs on oxidative stress-related biomarkers of 3D-photodamaged skin ($\bar{x}\pm S$).

Parameter	Normal group	Model group	Overexpressed group	Control group	Silenced group	Negative control group
MDA (*μ*mol/L)	4.06 ± 1.33	8.51 ± 2.11^#^	3.65 ± 1.06^&†$♫^	5.52 ± 1.09	8.49±1.62^∗∗^	5.61 ± 1.02
ROS (U/*μ*L)	9.09 ± 4.25	20.19 ± 5.31^#^	8.82 ± 3.05^&†$♫^	12.98 ± 2.71	19.65 ± 4.32^∗^	13.36 ± 2.62
SOD (U/mL)	121.35 ± 18.01	55.71 ± 12.72^#^	132.36 ± 16.25^&†$♫^	93.57 ± 9.11	59.01±12.15^∗∗^	92.26 ± 9.05
CAT (U/L)	22.35 ± 5.72	11.37 ± 3.57^#^	23.25 ± 5.53^&†♫^	19.17 ± 3.21	11.39±3.05^∗∗^	18.35 ± 3.33
GPX (U/mL)	79.91 ± 15.26	46.23 ± 7.32^#^	80.37 ± 13.52^&†$♫^	62.93 ± 7.08	45.93±7.25^∗∗^	62.05 ± 7.03
GSH (*μ*mol/L)	311.68 ± 42.33	91.99 ± 36.25^#^	315.52 ± 40.53^&†$♫^	237.53 ± 33.27	90.01±32.06^∗∗^	236.96 ± 33.55

Data are presented as mean ± standard deviation. ^#^*P* < 0.01 versus the normal group; ^&^*P* < 0.01 versus the model group; ^†^*P* < 0.01 versus the control group; ^$^*P* < 0.05 versus the silenced group; ^♫^*P* < 0.05 versus the negative control group; ^∗^*P* < 0.05 and ^∗∗^*P* < 0.01 versus the negative control group (Student's *t*-test).

## Data Availability

The data used to support the findings of this study are available from the corresponding author upon request.

## Abbreviations

UV: Ultraviolet
ROS: Reactive oxygen species
Nrf2: Nuclear factor erythroid 2-related factor 2
SKPs: Skin-derived precursors
*nrf2-over-*SKPs: SKPs with overexpressed *nrf2* expression
*si-nrf2-*SKPs: SKPs with silenced *nrf2* expression
3D: Three dimensional
FBs: Fibroblasts
KC: Keratinocytes
SOD: Superoxide dismutase
CAT: Catalase
GPX: Glutathione peroxidase
GSH: Glutathione
MDA: Malonaldehyde
IHC: Immunohistochemical
H&E: Hematoxylin and eosin
qRT-PCR: Quantitative reverse transcription PCR
ANOVA: Analysis of variance
PI3K: Phosphoinositide-3-kinase.

## Supplementary Materials

Supplementary Materials This figure primarily described the effect of nrf2-gene-modified SKPs against the UV-induced damage of the three-dimensional (3D) skin equivalent in vitro. Briefly, nrf2-overexpressed SKPs (emitting red fluorescence) and nrf2-silenced SKPs (emitting green fluorescence) were injected into 3D skin equivalents (3D skin equivalent was simultaneously built and identified by H&E staining and IHC analysis); then, simulated sunlight (UVA+UVB) was applied to 3D skin equivalents or not; finally, the alternations of histology, apoptosis, cellular protective genes, and antioxidant proteins in 3D skin equivalents were investigated. The above findings demonstrate that the protective effect of SKPs against UV-mediated damage is primarily via the PI3K/Akt-mediated activation of the Nrf2/HO-1 pathway, suggesting that SKPs may be a promising candidate for the treatment of photodermatoses. If you have any question, do not hesitate to contact me.
