# Supplementary figures and images for: Nrf2 Overexpression for the Protective Effect of Skin-Derived Precursors against UV-Induced Damage: Evidence from a Three-Dimensional Skin Model
Source: Oxid Med Cell Longev. 2019 Oct 14;2019:7021428. doi: 10.1155/2019/7021428 (PMC6815583; doi:10.1155/2019/7021428)

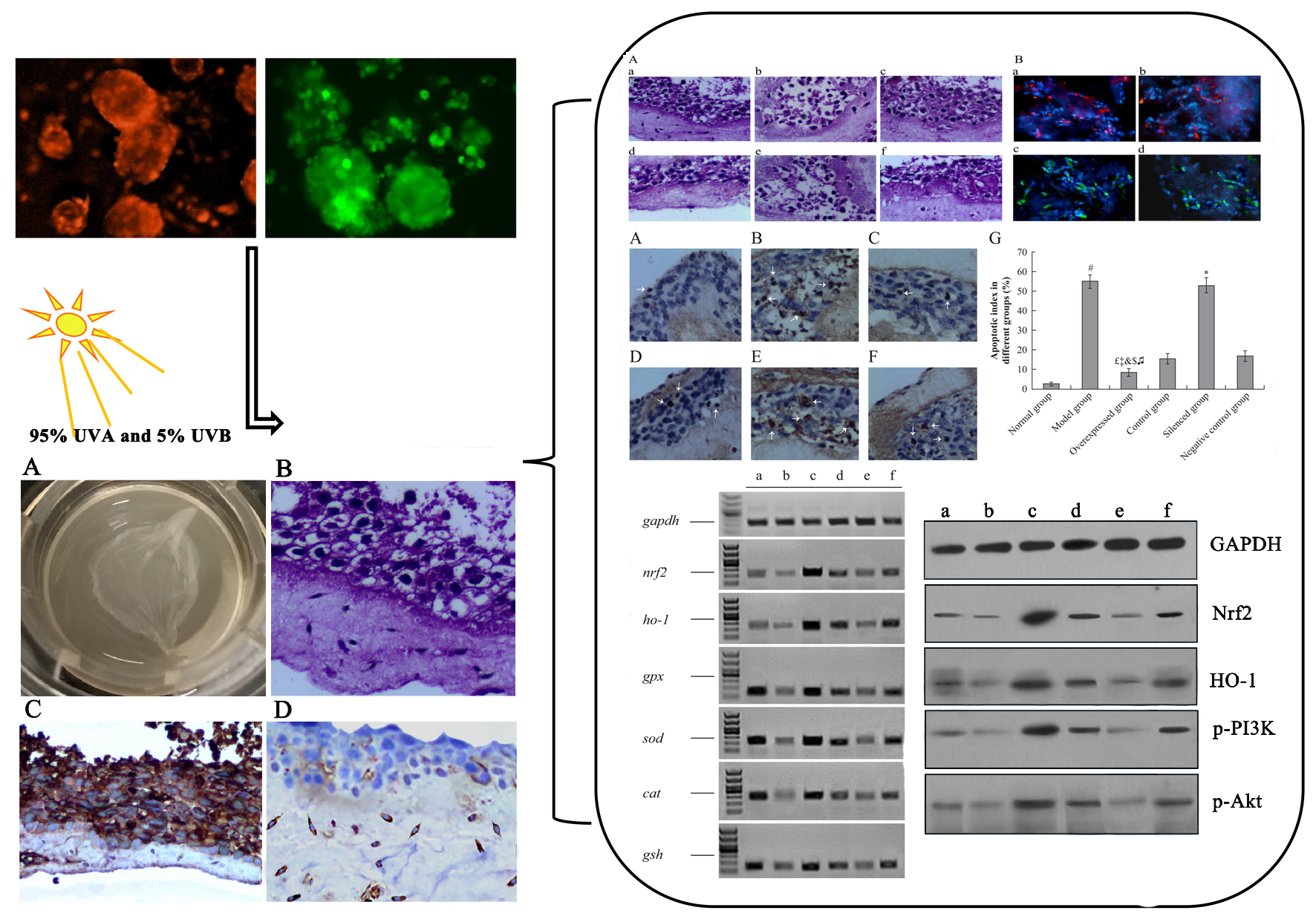

Supplement: Supplementary Materials — This figure primarily described the effect of nrf2-gene-modified SKPs against the UV-induced damage of the three-dimensional (3D) skin equivalent in vitro. Briefly, nrf2-overexpressed SKPs (emitting red fluorescence) and nrf2-silenced SKPs (emitting green fluorescence) were injected into 3D skin equivalents (3D skin equivalent was simultaneously built and identified by H&E staining and IHC analysis); then, simulated sunlight (UVA+UVB) was applied to 3D skin equivalents or not; finally, the alternations of histology, apoptosis, cellular protective genes, and antioxidant proteins in 3D skin equivalents were investigated. The above findings demonstrate that the protective effect of SKPs against UV-mediated damage is primarily via the PI3K/Akt-mediated activation of the Nrf2/HO-1 pathway, suggesting that SKPs may be a promising candidate for the treatment of photodermatoses. If you have any question, do not hesitate to contact me. [file 7021428.f1.jpg]
